# Hyperglycemia can delay left ventricular dysfunction but not autonomic damage after myocardial infarction in rodents

**DOI:** 10.1186/1475-2840-10-26

**Published:** 2011-04-06

**Authors:** Bruno Rodrigues, Kaleizu T Rosa, Alessandra Medeiros, Beatriz D Schaan, Patricia C Brum, Kátia De Angelis, Maria Cláudia Irigoyen

**Affiliations:** 1 Human Movement Laboratory, São Judas Tadeu University, São Paulo, Brazil; 2 Hypertension Unit, Heart Institute (InCor), Medical School of University of São Paulo, São Paulo, Brazil; 3 Federal University of São Paulo, Biosciences Department, Santos, SP, Brazil; 4 Endocrine Division, Clinical Hospital of Porto Alegre, Federal University of Rio Grande do Sul, Porto Alegre, Brazil; 5 School of Physical Education and Sports, University of São Paulo, São Paulo, Brazil; 6 Nove de Julho University, São Paulo, Brazil

## Abstract

**Background:**

Although clinical diabetes mellitus is obviously a high risk factor for myocardial infarction (MI), in experimental studies disagreement exists about the sensitivity to ischemic injury of an infarcted myocardium. Recently, our group demonstrated that diabetic animals presented better cardiac function recovery and cellular resistance to ischemic injury than nondiabetics. In the present study, we evaluated the chronic effects of MI on left ventricular (LV) and autonomic functions in streptozotocin (STZ) diabetic rats.

**Methods:**

Male Wistar rats were divided into 4 groups: control (C, n = 15), diabetes (D, n = 16), MI (I, n = 21), and diabetes + MI (DI, n = 30). MI was induced 15 days after diabetes (STZ) induction. Ninety days after MI, LV and autonomic functions were evaluated (8 animals each group). Left ventricular homogenates were analyzed by Western blotting to evaluate the expression of calcium handling proteins.

**Results:**

MI area was similar in infarcted groups (~43%). Ejection fraction and +dP/dt were reduced in I compared with DI. End-diastolic pressure was additionally increased in I compared with DI. Compared with DI, I had increased Na^+^-Ca^2+ ^exchange and phospholamban expression (164%) and decreased phosphorylated phospholamban at serine^16 ^(65%) and threonine^17 ^(70%) expression. Nevertheless, diabetic groups had greater autonomic dysfunction, observed by baroreflex sensitivity and pulse interval variability reductions. Consequently, the mortality rate was increased in DI compared with I, D, and C groups.

**Conclusions:**

LV dysfunction in diabetic animals was attenuated after 90 days of myocardial infarction and was associated with a better profile of calcium handling proteins. However, this positive adaptation was not able to reduce the mortality rate of DI animals, suggesting that autonomic dysfunction is associated with increased mortality in this group. Therefore, it is possible that the better cardiac function has been transitory, and the autonomic dysfunction, more prominent in diabetic group, may lead, in the future, to the cardiovascular damage.

## Introduction

Diabetes has been associated with an increased risk of cardiovascular abnormalities and microvascular complications. Although microvascular retinopathy and nephropathy are associated with a high degree of morbidity, the increased mortality in patients with diabetes is primarily a consequence of cardiovascular disease [1]. In fact, clinical studies have demonstrated that diabetes, with consequent cardiomyopathy and cardiovascular neuropathy, is an independent risk factor for cardiovascular disease and is associated with a 2- to 4-fold increased risk of coronary heart disease [2].

In this regard, experimental data have demonstrated that hyperglycemia in diabetic animals leads to changes in the heart that contribute to injury during and following an ischemic event; however, the response of an uncontrolled hyperglycemic diabetic heart to ischemic injury remains controversial [3-5]. Experimental studies [6-8] using an ischemia/reperfusion protocol have shown that hearts from streptozotocin (STZ) diabetic rats that undergo a period of no-flow ischemia have a reduced myocardial infarction (MI) area and recovered ventricular function significantly better than nondiabetic hearts, indicating a possible cardioprotective role of hyperglycemia. In fact, the exposure to short periods of abnormally higher glucose medium or diabetes has been found to protect the heart against a variety of pathological insults, including ischemia, hypoxia, and calcium overload [9,10].

However, most studies reported in the literature have been conducted using an ischemia/reperfusion model, thus it has not been possible to verify whether chronic hyperglycemia can protect the diabetic heart against permanent coronary ligation. Recently, our group demonstrated that after 15 days of MI, diabetic animals had reduced heart fibrosis, improved systolic function, and reduced infarct size. In accordance with this study, it is possible that these data may have indicated the final pathway promoted by a positive balance in regulatory genes related to programmed cell survival, reduced inflammatory cytokines, and increased utilization of glucose as an energy substrate [9]. However, other important risk factors, such as cardiovascular autonomic neuropathy, and influences on the mortality rate were not evaluated in this study.

Changes in critical processes that regulate intracellular calcium concentration and signaling have been a hallmark of cardiomyopathy and heart failure. Alterations in the expression and activity of cardiac proteins that participate in the calcium handling, (e.g., calcium pump ATPase of sarcoplasmic reticulum (SERCA2), dephosphorylated phospholamban (PLN), which respectively decreases the affinity of SERCA2 for calcium, and sarcolemmal sodium calcium exchanger (NCX), which mediates calcium efflux from the cell [11] have been shown to occur in cardiomyopathy of STZ diabetic rats [12] and after MI [13]. However, the impact of association between STZ diabetes and MI in the expression of calcium handling proteins remains unknown. In this sense, the aim of the present study was to investigate the effects of hyperglycemia induced by STZ diabetes in left ventricular (LV) dysfunction, net balance of regulatory proteins involved in intracellular calcium homeostasis, autonomic dysfunction and mortality rate, in rats that underwent 90 days of MI.

## Methods

### Animals and Groups

Experiments were performed in adult male Wistar rats (~240 g) from the Animal House of the University of São Paulo, São Paulo, Brazil. Rats were fed standard laboratory chow and water *ad libitum*. The animals were housed in collective polycarbonate cages in a temperature-controlled room (22°C) with a 12-hour dark-light cycle (light 07:00-19:00 h). The experimental protocol was approved by the institutional animal care and use committee of the Medical School of the University of São Paulo, and this investigation was conducted in accordance with the previously described [14]. Rats were randomly assigned to control (C, n = 15), diabetes (D, n = 16), myocardial infarction (I, n = 21), and diabetes + myocardial infarction (DI, n = 30).

### Diabetes Induction

Experimental diabetes was induced by intravenous injection of 50 mg/kg STZ (Sigma Chemical Co., St. Louis, MO) dissolved in citrate buffer (pH 4.2). Rats were fasted overnight before STZ injection. Control rats were injected with buffer only (10 mM citrate buffer, pH 4.5). Forty-eight hours after STZ injection, diabetes was confirmed by blood glucose levels above 200 mg/dL.

### Myocardial Infarction

Fifteen days after diabetes induction or buffer injection, anesthetized rats (80 mg/kg Ketamine and 12 mg/kg Xylazine, i.p.) underwent surgical occlusion of the left coronary artery, which resulted in myocardial infarction, as previously described elsewhere [9,13]. Briefly, a left intercostal thoracotomy was performed, the third intercostal space dissected, and the heart exposed. The left anterior descending coronary artery was occluded with a single nylon (6.0) suture at approximately 1 mm distal to the left atrial appendage. The chest was closed with silk suture.

The animals were maintained in the ventilator until recovery. All rats received antibiotics (penicillin, 20,000 U) and Tramadol (20 mg/kg, every 6 h). Eight animals from each group were evaluated 90 days after myocardial infarction induction (105 days after diabetes induction) to perform the following experimental protocols. The whole sample (n = 75) was evaluated for mortality, an assessment that started after myocardial infarction surgery, excluding the influence of anesthesia or surgical procedure stress.

### Noninvasive Evaluation of Left Ventricular Function: Echocardiographic Measurements

Echocardiography was performed by a double-blinded observer, under the guidelines of the American Society of Echocardiography, 2 days (initial evaluation) and 90 days (final evaluation) after myocardial infarction. Rats were anesthetized (80 mg/kg Ketamine and 12 mg/kg Xylazine), and images were obtained with a 10-14 mHz linear transducer in a SEQUOIA 512 (ACUSON Corporation, Mountain View, CA) for measurements of morphometric parameters: left ventricular (LV) mass (corrected by body weight) and LV end-diastolic diameter (LVEDD); systolic function parameters: ejection fraction (EF) and velocity of circumferential fiber shortening (VCF); diastolic function parameters: LV isovolumetric relaxation time (IVRT) and peak E deceleration time (EDT) corrected by the square root of R-R interval, because EDT and IVRT are HR dependent; and global function: myocardial performance index (MPI). Echocardiographic parameters were measured as described in detail elsewhere [9,13].

The infarction area was delimited, which led us to analyze the movement of the LV walls. Regions with systolic shortening classified as absent or with paradoxical movement were considered infarcted. The infarcted area (in %) was thus measured as the ratio of these regions by the total area of LV walls [9,13,15].

### Autonomic Evaluations

One day after the final echocardiographic evaluation, 2 catheters filled with 0.06 mL of saline were implanted into the femoral artery and femoral vein of the anesthetized rats (80 mg/kg Ketamine and 12 mg/kg Xylazine, IP). Twenty-four hours later, the arterial cannula was connected to a strain-gauge transducer (Blood Pressure XDCR, Kent Scientific, USA), and arterial pressure (AP) signals were recorded over a 30-minute period in conscious animals by a microcomputer equipped with an analog-to-digital converter board (WinDaq, 2-kHz, DATAQ, Springfield, OH). The recorded data were analyzed on a beat-to-beat basis to quantify changes in mean AP (MAP) and heart rate (HR) [13,15].

Sequential bolus injections (0.1 mL) of increasing doses of phenylephrine (0.25 to 32 μg/kg) and sodium nitroprusside (0.05 to 1.6 μg/kg) were given to induce increases or decreases in MAP pressure responses (for each drug), ranging from 5 to 40 mm Hg. Baroreflex sensitivity expressed as bradycardic (BR) and tachycardic (TR) responses in beats per minute per millimeter of mercury, as described elsewhere. The overall variability of the pulse interval (PI) in the time domain was assessed by the standard deviation (SD) of the time series [13,15].

### Invasive Evaluation of Left Ventricular Function

One day after the final autonomic evaluation, LV function was also measured invasively in anesthetized rats (pentobarbital sodium, 40 mg/kg). One catheter of PE-50 was inserted into the right carotid artery and advanced into the LV. Ventricular pressure signals were measured with a transducer and conditioner (Blood Pressure XDCR, Kent^© ^Scientific, USA) and digitally recorded (5 min) with a data acquisition system (WinDaq, 2-kHz, DATAQ, Springfield, OH). The recorded data were analyzed on a beat-to-beat basis to quantify changes in LV pressure. The following indices were obtained: heart rate (HR), LV systolic pressure (LVSP), LV end-diastolic pressure (LVEDP), and maximum rate of LV pressure rise and fall (+dP/dt and -dP/dt), as previously described [13,16].

### Western Blot Analysis

Left ventricular homogenates were analyzed by Western blotting to evaluate the expression of sarcoplasmic reticulum calcium ATPase pump (SERCA2), phospholamban (PLN), phosphorylated - PLN at serine 16 (phospho-ser^16^-PLN), phosphorylated - PLN at threonine 17 (phospho-thr^17^-PLN), phosphatase protein 1 (PP1), and sodium calcium exchanger (NCX), as described elsewhere [17]. Mouse monoclonal antibodies to SERCA2 (1:2500), PLN (1:500), and NCX (1:2000) were obtained from Affinity BioReagents (Golden, CO); rabbit polyclonal antibody to protein phosphatase type 1 (PP1, 1:1000) and protein phosphatase type 2 A (PP2A, 1:1000) were obtained from Upstate (Lake Placid, NY); phospho-ser^16^-PLN (1:5000) and phospho-thr^17^-PLN (1:5000) were obtained from Badrilla (Leeds, UK). Glyceraldehyde-3-phosphate dehydrogenase (GAPDH, 1:2000) was obtained from Advanced Immunochemical (Long Beach, CA). Targeted bands were normalized to cardiac GAPDH.

### Statistical Analysis

Data are reported as means ± SEM. Two-way and repeated measures ANOVA were used to compare groups followed by the Student-Newman-Keuls post-test. The Kaplan-Meier method was used to determine the survival curves, which were compared by using the log-rank test. Pearson correlation was used to study associations between variables. Significance level was established at p < 0.05.

## Results

### Animals

At the beginning of the protocol, body weight was similar between the groups (~230 ± 15 g). After 90 days of MI, STZ-diabetic rats had a reduced body weight (D: 213 ± 10 and DI: 211 ± 10 g) compared with normoglycemic rats (C: 508 ± 3 and I: 471 ± 9 g). Glycemia was increased in STZ-diabetic rats (D: 400 ± 27 and DI: 371 ± 37 mg/dL) in comparison with normoglycemics (C: 90 ± 3 and I: 96 ± 3 mg/dL).

### Left Ventricular Function: Noninvasive and Invasive Evaluations

The myocardial infarction akinetic area (MI area) measured by echocardiography was similar between infarcted groups at the initial (I: 40 ± 3 and DI: 41 ± 3% of LV wall) and final (I: 47 ± 2 and DI: 40 ± 4% of LV wall) evaluations. There was a correlation between the MI area evaluated by echocardiography and Masson's trichrome stain histological method (r = 0.90, p < 0.0001).

The noninvasive left ventricular function parameters are shown in Table 1. Initial evaluation, performed 2 days after the myocardial infarction surgery, showed that LV mass, IVRT, EDT, and MPI were similar between the experimental groups. In contrast, increased LV chamber (LVEDD), reduced EF, and VCF were observed in the infarcted groups (I and DI) compared with noninfarcted (C and D) rats at the initial evaluation.

**Table 1:** Initial and final echocardiographic measurements in control (C), diabetes (D), myocardial infarction (I), and diabetes + myocardial infarction (DI) groups.

Parameters		C	D	I	DI
***Morphometric***					
**LV mass (g/kg)**	Initial	1.02 ± 0.02	0.95 ± 0.01	1.05 ± 0.04	1.04 ± 0.01
	Final	1.11 ± 0.03	0.95 ± 0.02	1.18 ± 0.06#	1.16 ± 0.06
**LVEDD (cm)**	Initial	0.65 ± 0.01	0.63 ± 0.01	0.75 ± 0.01*†	0.77 ± 0.03*†
	Final	0.71 ± 0.02	0.71 ± 0.02	0.85 ± 0.03*†	0.86 ± 0.03*†
***Systolic***					
**EF (%)**	Initial	74 ± 2	80 ± 1	48 ± 3*†	51 ± 3*†
	Final	71 ± 1	61 ± 2#	42 ± 3*†	55 ± 5*‡
**VCF (circ/s) (10^-4^)**	Initial	56 ± 3	54 ± 2	38 ± 2*	38 ± 3*
	Final	50 ± 3	38 ± 4#*	34 ± 2*	43 ± 1‡
***Diastolic***					
**IVRT (ms)**	Initial	1.81 ± 0.07	1.96 ± 0.05	1.81 ± 0.09	1.94 ± 0.08
	Final	1.79 ± 0.05	2.07 ± 0.07*	2.03 ± 0.05*	2.16 ± 0.15*
**EDT (ms)**	Initial	1.87 ± 0.11	1.95 ± 0.10	1.97 ± 0.10	1.83 ± 0.09
	Final	1.75 ± 0.07	2.20 ± 0.09#*	2.13 ± 0.09*	2.45 ± 0.25#*
***Global Function***					
**MPI**	Initial	0.39 ± 0.01	0.41 ± 0.03	0.46 ± 0.03	0.44 ± 0.03
	Final	0.34 ± 0.03	0.50 ± 0.02*	0.57 ± 0.04*	0.45 ± 0.01*‡

Values are expressed as mean ± SEM. LV mass - Left ventricular mass corrected by body weight; LVEDD - Left ventricular end-diameter during diastole; EF - Ejection fraction; VCF - Velocity of circumferential fiber shortening; EDT- Peak E desacceleration time; IVRT- Left ventricular isovolumetric relaxation time; MPI - Myocardial performance index. # p < 0.05 vs. initial evaluation; * p < 0.05 vs. C; † p < 0.05 vs. D; ‡ p < 0.05 vs. I (n = 8 for each group).

Final echocardiographic evaluation (90 days after myocardial infarction, 105 days after STZ induction) showed that LV mass was not different in D rats in relation to the C; however, the I group had an increase compared with the initial evaluation. Moreover, diastolic dysfunction was present in D, I, and DI compared with C rats and in the diabetic groups (D and DI) compared with that at the initial evaluation. The D group had reduced systolic function evaluated by EF and VCF in relation to the initial evaluation and C group (VCF). Myocardial infarction groups (I and DI) demonstrated systolic dysfunction compared with the C group. However, DI animals showed attenuation of systolic dysfunction, as demonstrated by an increase in EF and VCF compared with I rats at the final evaluation. MPI was increased in the final evaluations of the D, I, and DI groups in comparison with the C group. Parallel with these results, DI had reduced MPI compared with the I group, indicating lower global ventricular dysfunction in these animals.

Invasive LV function data, performed after the final echocardiographic evaluation, demonstrated that the LVSP was reduced in D, I, and DI groups compared with that in the C group (92 ± 3, 113 ± 4, 93 ± 4 vs. 134 ± 5 mmHg, respectively). Furthermore, STZ-diabetic animals (D and DI) had additional impairment in LVSP compared with I animals. As expected, LVEDP was markedly increased in both I (20 ± 2 mmHg) and DI (12 ± 3 mmHg) rats compared with D and C rats (6 ± 1 and 5 ± 0.3 mmHg, respectively). However, the DI group had attenuated LVEDP compared with the I group. Ventricular function was also estimated by +dP/dt (inotropic index) and -dP/dt (lusitropic index). Experimental groups (D, I, and DI) exhibited diastolic dysfunction evaluated by -dP/dt compared with the C group (D: -4315 ± 473, I: -3208 ± 481, DI: -4030 ± 484 vs. C: -7186 ± 169 mmHg/sec). Similarly, D, I, and DI animals displayed a reduction in +dP/dt inotropic index (6567 ± 415, 4301 ± 457 and 5702 ± 325 mmHg/sec, respectively) compared with C animals (9445 ± 420 mmHg/sec). However, it is important to point out that the DI group had attenuated systolic dysfunction, also evidenced by invasive measurements (+dP/dt), compared with I rats.

Invasive LV function data paralleled echocardiography findings, as observed by the positive correlation obtained between LVEDD and LVEDP (r = 0.85; p < 0.0005), suggesting that increased diastolic diameter was associated with higher LVEDP values. In addition, a positive correlation was also obtained between EF and +dP/dt (r = 0.82; p = 0.0007).

### Hemodynamic and Autonomic Function

**Figure 1:**
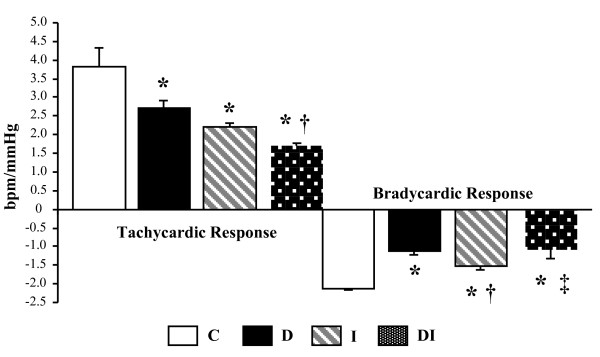
**Baroreflex sensitivity estimated by bradycardic (BR) and tachycardic responses (TR) from control (C), diabetes (D), myocardial infarction (I) and diabetes + myocardial infarction (DI) rats (n = 8 for each group)**. * p < 0.05 vs. C; † p < 0.05 vs. D; ‡ p < 0.05 vs. I.

Hemodynamic and autonomic evaluations are presented in Table 2. Diabetic groups (D and DI) had a reduction in mean arterial pressure (MAP) and heart rate (HR) compared with that in nondiabetic groups (C and I). In addition, D, I, and DI animals had a reduction in systolic arterial pressure in comparison with the C group; however, STZ-diabetic (D and DI) rats evidenced an additional reduction of this parameter compared to C and I. Baroreflex sensitivity, evaluated by tachycardic (TR) and bradycardic responses (BR), was worsened in all experimental groups in comparison with C. Therefore, D and DI displayed an additional reduction in BR compared with C and I rats, as observed in the Figure 1.

**Table 2:** Hemodynamic measurements and pulse interval variability in control (C), diabetes (D), myocardial infarction (I), and diabetes + myocardial infarction (DI) groups.

Parameter/Group	C	D	I	DI
***Hemodynamic***				
**SAP (mm Hg)**	129 ± 2	109 ± 3*	117 ± 3*†	106 ± 2*‡
**DAP (mm Hg)**	92 ± 2	83 ± 2*	90 ± 3	87 ± 2*
**MAP (mm Hg)**	111 ± 2	96 ± 3*	104 ± 3	99 ± 2*
**HR (bpm)**	352 ± 12	302 ± 7*	342 ± 6	307 ± 19*
***Pulse Interval Variability***				
**SD (bpm)**	34 ± 3	22 ± 4*	24 ± 2*	13 ± 1*†‡

Values are expressed as mean ± SEM. SAP - Systolic arterial pressure; DAP - Diastolic arterial pressure; MAP - Mean arterial pressure; HR - Heart rate; SD - Standard deviation. * p < 0.05 vs. C; † p < 0.05 vs. D; ‡ p < 0.05 vs. I (n = 8 for each group).

Pulse interval variability, evaluated in time domain by standard deviation of pulse interval (SD), was reduced in all experimental animals compared with C, with additional impairment in the DI group compared with that in D and I (Table 2).

### Expression of Regulatory Proteins Involved in Intracellular Calcium Homeostasis

**Figure 2:**
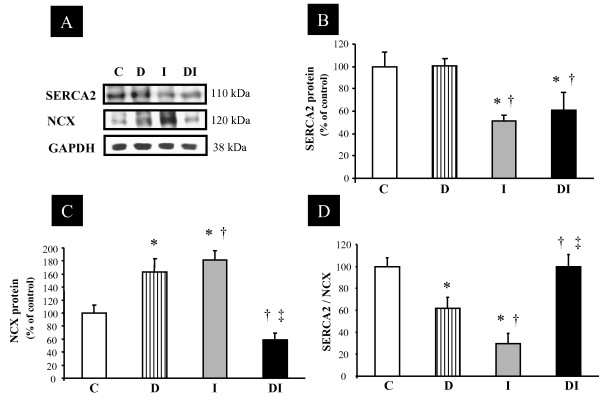
**Expression levels of regulatory proteins related to intracellular calcium homeostasis from control (C), diabetes (D), myocardial infarction (I), and diabetes + myocardial infarction (DI) rats (n = 8 for each group)**. Targeted bands were normalized to cardiac GAPDH. **A) **Representative blots of SERCA2, Na^+^-Ca^2+ ^exchanger (NCX) and GAPDH; **B) **SERCA2; **C) **NCX; and **D) **SERCA2/NCX ratio. * p < 0.05 vs. C; † p < 0.05 vs. D; ‡ p < 0.05 vs. I.

GAPDH protein levels remained unchanged among all groups studied and were used to normalize regulatory protein expression involved in calcium homeostasis. SERCA2 expression levels were reduced in both infarcted groups (I and DI) compared with C and D (Figures 2A and 2B). In addition, positive correlations were found between SERCA2 and EF (r = 0.85; p < 0.001) and SERCA2 and +dP/dt (r = 0.87; p < 0.001). NCX expression levels were increased in D and I groups compared with C; however, DI rats had NCX expression levels reduced in comparison with that in D and I rats (Figures 2A and 2C). The SERCA2/NCX ratio was reduced in D and I animals in comparison with C, and increased in DI in comparison with D and I animals (Figure 2D).

**Figure 3:**
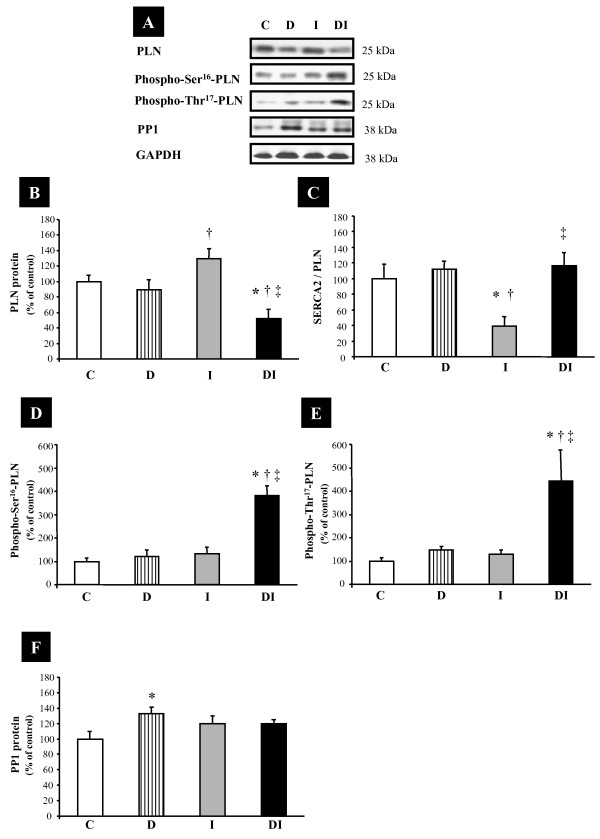
**Expression levels of intracellular calcium efflux mediators from control (C), diabetes (D), myocardial infarction (I), and diabetes + myocardial infarction (DI) rats (n = 8 for each group)**. Targeted bands were normalized to cardiac GAPDH. **A) **Representative blots of Phospholamban (PLN), Phosho-Ser^16^-PLN, Phosho-Thr^17^-PLN, PP1, and GAPDH; **B) **PLN; **C) **SERCA2/PLN ratio; **D) **Phosho-Ser^16^-PLN normalized to total PLN; **E) **Phosho-Thr^17^-PLN normalized to total PLN; and **F) **PP1. * p < 0.05 vs. C; † p < 0.05 vs. D; ‡ p < 0.05 vs. I.

We additionally evaluated the expression of PLN, a phosphoprotein that regulates the apparent calcium affinity of SERCA2, and phosphorylated PLN in both Ser^16 ^and Thr^17 ^residues. PLN expression levels were increased in group I compared with that in D, and reduced in DI compared with that in C, D, and I groups (Figures 3A and 3B). Additionally, the SERCA2/PLN ratio, an important index of sarcoplasmic reticulum calcium uptake capacity, was reduced in I animals compared with C, D, and DI animals (Figure 3C). The expression levels of both phospho-ser^16^-PLN and phospho-thr^17^-PLN were increased only in DI animals compared with C, D, and I rats (Figures 3A, D and 3E), indicating higher activation of SERCA2 pump and better calcium reuptake by sarcoplasmic reticulum in this group. PP1 expression, a PLN phosphorylation regulator, was increased in the D group compared with that in C; however, although the increase observed in I and DI was discrete, no statistical changes were evidenced (Figures 3A and 3F).

### Mortality Rate

Total mortality rate evaluation (Kaplan-Meier survival curve) showed that groups I (13 deaths among 21 animals, 62% of mortality) and DI (22 deaths among 30 animals, 73% of mortality) had a higher mortality rate compared with C (no deaths) and D groups (8 deaths among 16 animals, 50% of mortality). However, the DI group had an increased mortality rate compared with I group.

## Discussion

The key finding of the present study was that the ventricular dysfunction determined by myocardial infarction was attenuated in diabetic rats, an attenuation that was associated with improved net balance of regulatory proteins that participate in the cardiac calcium handling. In opposition, autonomic dysfunction was more prominent in DI compared with I. This alteration may have induced an increase in the mortality rate, as observed in DI compared with normoglycemic MI animals.

Epidemiological studies have shown that the incidence of microvascular, macrovascular, and autonomic complications in diabetic patients is directly associated with the degree of hyperglycemia [18]. Although intensive glucose control in patients with diabetes has been acknowledged to reduce the risk of vascular complications, the evidence that glycemic control results in a reduction in cardiovascular disease is limited. Several prospective intervention studies published in the past 2 years have investigated whether intensive glucose control to near-normoglycemia reduces cardiovascular events and mortality in individuals with diabetes and have produced conflicting results, especially with regard to risk of cardiovascular events [19-21].

In addition to the clinical studies and trials, experimental designs also have reported controversial data about the role of hyperglycemia in the mechanisms involved in cardioprotection in animals. Several studies have shown that hearts from diabetic rats are equally [22] or more [5,23] sensitive to ischemic injury. In contrast, previous reports have demonstrated that glucose supply plays a critical cardioprotective role in cardiac responses during acute ischemia. These findings suggest that higher glucose delivery to the heart may improve tolerance to ischemic stress, leading to changes in signaling mechanisms involved in cardioprotection [24,25]. Recently, Chu et al. [8] provided evidence that diabetes induced by Aloxan and resultant hyperglycemia, though detrimental to global cardiac function and associated with a poorer prognosis, was cardioprotective against myocardial ischemic-reperfusion injury in pigs in the short-term. The authors observed a pronounced reduction in the myocardial infarction area in diabetic animals, likely being a result of increased availability and use of glucose, the heart's preferred energy substrate in times of stress.

In fact, some studies have demonstrated that the reduction in myocardial infarction size [26] is determined by the diminished number of dead myocytes in diabetic animals [27], and would be the key point to the higher resistance of diabetic rats to ischemic injury. In contrast with the results in the literature and data from our group [9], the MI area was similar between I and DI groups in the present study. This discrepancy in data may be the result of the prolonged time of diabetes and MI investigated here. Thus, the higher resistance of the diabetic heart cannot be explained by the reduction in the myocardial infarction akinetic area in the present study.

### Morphological and Functional Responses to Ischemic Injury

Myocardial remodeling after MI is usually characterized by compensatory hypertrophy of myocytes, ventricular dilatation and increased interstitial collagen deposition and fibrosis [5]. In our study, LVEDD was increased in infarcted compared with noninfarcted groups; however I and DI groups had similar results of LV mass and LVEDD, highlighting the fact that STZ-diabetes had no influence on cardiac remodeling after MI. To the contrary, Bäcklund et al. [5] showed that 12 weeks after MI surgery cardiomyocyte apoptosis was associated with LV enlargement and increased cardiac fibrosis in diabetic animals. However, it is possible that the prolonged time of STZ-diabetes (4 weeks) before the MI surgery, in contrast with our study (15 days), has been the responsible for discrepancy in the results.

To further investigate the mechanisms underlying the improved tolerance to ischemia of diabetic rats, we evaluated the cardiac function of the experimental groups. Our data indicate that diabetes partially attenuated the ventricular dysfunction caused by myocardial ischemia (DI animals compared with I animals). In fact, DI animals had improved systolic and diastolic function indexes, paralleled by reduced MPI, an index that represents global myocardial stress [16]. These data corroborate previous findings of our group where 15 days after MI, STZ-diabetic rats had attenuation of cardiac dysfunction compared with the normoglycemic rats. Interestingly, this attenuation was associated with an improved net balance of inflammatory cytokines and regulatory genes that participate in cardiac cellular survival [9].

### Molecular Changes

Because cardiac dysfunction observed in cardiomyopathy and heart failure is strongly associated with the expression profile of the cardiac proteins related to intracellular calcium handling [11], we evaluated the SERCA2, PLN, phospho-ser^16^-PLN, phospho-thr^17^-PLN, PP1, and NCX. It was previously reported that in heart failure cardiac dysfunction is in part a consequence of changes in intracellular calcium homeostasis, which may be related to an altered expression, function, or regulation of SERCA2 [11,28]. In the present study, although the MI groups (I and DI) displayed a reduction in SERCA2 expression, the DI group had a decrease in NCX and PLN proteins, resulting in increased SERCA2/NCX and SERCA2/PLN ratios in comparison with those in the I group. This finding may indicate a relative increase in SERCA2 pump function and increased available calcium in the cytoplasm in diabetic animals that undergo MI, if compared with normoglycemic MI rats. Likewise, increased phosphorylation of PLN in both serine^16 ^and threonine^17 ^in DI rats reinforces the possibility that SERCA2 function is better preserved in these experimental animals. Indeed, the phosphorylation of PLN in both residues is involved in an increased calcium reuptake by sarcoplasmic reticulum, although the increase in PLN phosphorylation at Thr^17 ^may be interpreted, in some cases, as one of the latest compensatory mechanisms activated when cardiac function is deteriorated, as observed in acidosis and reperfusion periods [29].

In the present study, because the molecular analyses were performed in all LV homogenates to quantify the expression of proteins by Western blot, it is possible that the compensatory mechanisms involving calcium regulation in the noninfarcted area might have influenced the results. However, it is necessary to consider that not only the myocardial infarction area, but also LV mass were similar between the infarcted groups (I and DI). These findings suggest that experimental diabetes is associated with the activation of endogenous cardioprotective mechanisms, which successfully remain after 90 days of myocardial ischemia attenuating the remaining LV dysfunction in the DI group.

### Autonomic Impairment and Mortality Rate

Independently of the mechanism involved in the preserved left ventricular function observed in DI rats, it was expected that the mortality rate would be reduced in this group compared with the I group. However, DI animals evidenced an increase in mortality rate 90 days after myocardial infarction compared with the I group, suggesting that other mechanisms and dysfunctions might be influencing the survival rate of the diabetic animals. Possible mechanisms, all involving the metabolic characteristic of the diabetic state, have been previously suggested: long duration of the diabetes, severity of hyperglycemia [30], high levels of circulating free fatty acids, and decreased glucose utilization by cardiomyocytes [31] which contribute to contractile derangement [32] and higher arrhythmia susceptibility [33]. In fact, van den Brom et al., [34], by using positron emission tomography and echocardiography, evidenced increases in myocardial fatty acid oxidation with a concomitant decrease of insulin-mediated myocardial glucose utilization in early diabetic cardiomyopathy. In addition, the authors observed that these metabolic alterations were associated with impaired myocardial function.

Cardiovascular autonomic neuropathy has been considered an important determinant of mortality in diabetic patients independently of other known risk factors [35]. In the present study, more prominent autonomic dysfunction was observed in the DI group compared with that in I animals. This evidence may indicate that autonomic dysfunction, previously described by us [36,37], could be influencing the mortality observed in D and mainly in DI rats. In accordance with these results, our group previously demonstrated that sedentary STZ diabetic rats had accentuated autonomic dysfunction and consequently a higher mortality rate (~49%) compared with control rats. Additionally, 8 weeks of aerobic exercise training attenuated the autonomic dysfunction and reduced the mortality levels in the trained group to 16% [36]. For this reason, new therapeutic strategies have been studied in diabetes management. Shyu et al., [38] described the isolation of pancreatic endocrine precursor cells, from adult human pancreatic tissue, and the redifferentiation of insulin-secreting β-like cells from in vitro-cultured insulin-producing cell precursors. These data emphasize that surgically resected pancreatic tissue may represent an alternative source of functional insulin-producing cells and to minimize the cardiovascular dysfunctions of diabetes.

### Study Limitations

Some possible limitations of the present investigation deserve comment. First, because the MI animals (I and DI) started the protocol with similar values for MI area and LF function and necropsy evaluations were not performed in the animals that died during the 90 days of the protocol, the prediction of survival (or mortality) based on these parameters becomes difficult and is limited to methods used in this study. Second, the hemodynamic and autonomic parameters were evaluated at the end of the protocol, and comparisons were made by including a control group in the experimental design. Indeed, the direct method for recording blood pressure depends on the catheterization of arterial vessels that are functional during a small time period. Consequently, the biological signals were recorded only at the end of the experimental period, leading to the lack of baseline values in the same animals at the start of the study.

## Conclusion

We have demonstrated that the left ventricular dysfunction in diabetic animals was attenuated after 90 days of myocardial infarction. This is probably a compensatory mechanism associated with metabolic and molecular adjustments of the cardiac tissue, where the end point was a better profile of calcium handling proteins. However, this positive adaptation was not able to reduce the mortality rate of DI animals. These data suggest that other mechanisms, as well as cardiovascular autonomic dysfunction identified in this study, is associated with mortality rate in these animals. It is important to emphasize that in the present investigation, the time course of diabetes and MI was determined and well knows, in contrast with that observed in clinical situations. Therefore, it is possible that the better cardiac function has been transitory in DI rats, and the autonomic dysfunction, more prominent in diabetics, may lead, in the future, to cardiovascular damage, as observed in diabetic patients after an ischemic event.

## List of abbreviations

BR: bradycardic response; DAP: diastolic arterial pressure; +dP/dt: maximum rate of left ventricular pressure rise; -dP/dt: maximum rate of left ventricular pressure fall; EDT: peak E wave deceleration time; EF: ejection fraction; GAPDH: glyceraldehyde-3-phosphate dehydrogenase; HR: heart rate; IVRT: left ventricular isovolumetric relaxation time; LV: left ventricle; LV mass: left ventricular mass corrected by body weight; LVEDD: left ventricular end-diastolic diameter; LVEDP: left ventricular end-diastolic pressure; LVSP: left ventricular systolic pressure; MAP: mean arterial pressure; MI: myocardial infarction; MPI: myocardial performance index; NCX: sodium calcium exchanger; phospho-ser16-PLN: phosphorylated phospholamban at serine 16; phospho-thr17-PLN: phosphorylated phospholamban at threonine 17; PI: pulse interval; PLN: dephosphorylated phospholamban; PP1: phosphatase protein 1; SAP: systolic arterial pressure; SD: standard deviation; SEM: standard error of mean; SERCA2: calcium pump ATPase of sarcoplasmic reticulum; STZ: streptozotocin; TR: tachycardic response; VCF: velocity of circumferential fiber shortening.

## Competing interests

The authors declare that they have no competing interests.

## Authors' contributions

BR designed, performed, and coordinated the experiments, as well as prepared the manuscript. KTR carried out the echocardiographic measurements. AM participated in the Western blot analysis. BDS helped to draft the manuscript. PCB participated in the data discussion and helped to draft the manuscript. KDA participated in the design, data discussion and helped to draft the manuscript. MCI conceived the study and participated in its design and coordination. All authors have read and approved the final manuscript.
